# Supplementary material for: Gain and loss of function variants in EZH1 disrupt neurogenesis and cause dominant and recessive neurodevelopmental disorders
Source: Nat Commun. 2023 Jul 11;14:4109. doi: 10.1038/s41467-023-39645-5 (PMC10336078; doi:10.1038/s41467-023-39645-5)
Supplement: Supplementary file 1 — Supplementary Information [file 41467_2023_39645_MOESM1_ESM.pdf]

SUPPLEMENTARY INFORMATION FOR

Gain and loss of function variants in EZH1 disrupt neurogenesis and cause dominant and recessive neurodevelopmental disorders

Included in this file are:

Supplementary Fig. 1
Supplementary Fig. 2
Supplementary Fig. 3
Supplementary Fig. 4
Supplementary Fig. 5
Supplementary Fig. 6
Supplementary Fig. 7
Supplementary Fig. 8
Supplementary Table 1
Supplementary Table 2
Supplementary Table 3

Supplementary Figure 1

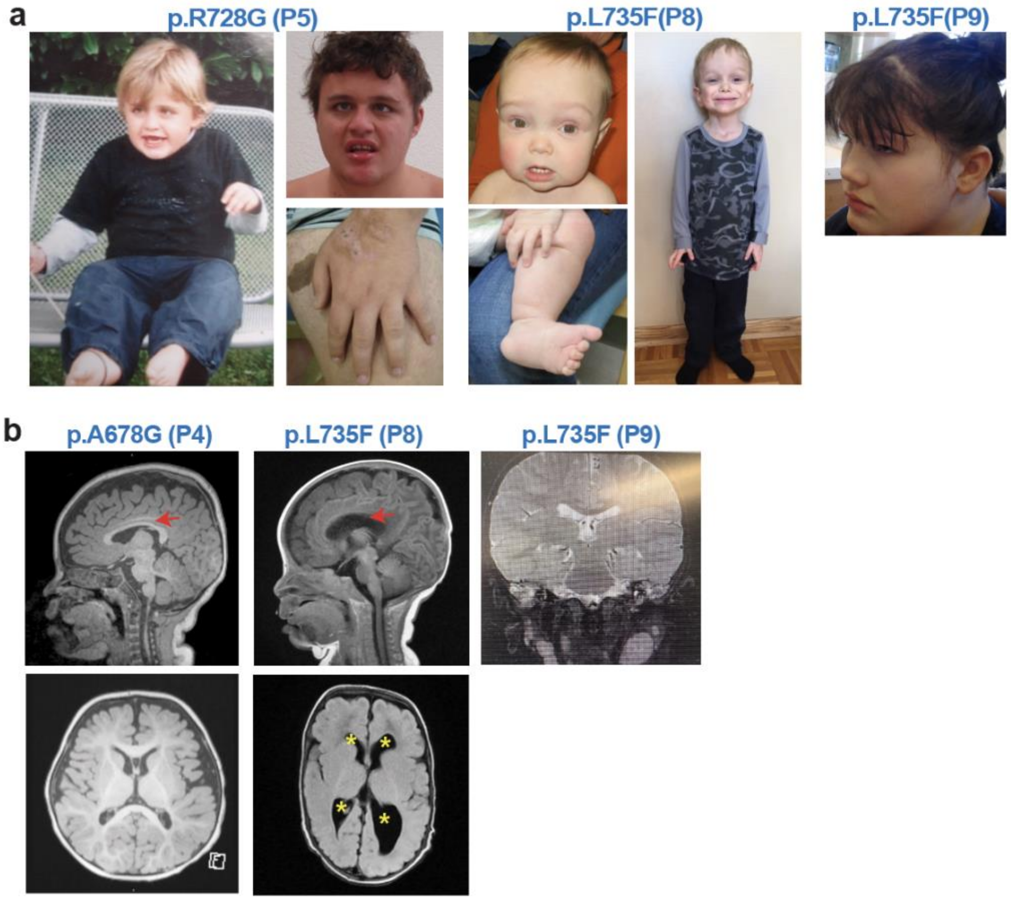

Supplementary Figure 1: Photos and brain MRIs of affected individuals carrying *EZH1* heterozygous and biallelic variants.
a, Face and body photos of patients with *EZH1* heterozygous missense variants at various ages. **b,** Brain MRIs, sagittal (up) and axial (down), of three patients with the indicated heterozygous variant. Red arrows point to the corpus callosum, while the yellow asterisks mark the enlarged lateral ventricles in P8 MRI.

Supplementary Figure 2

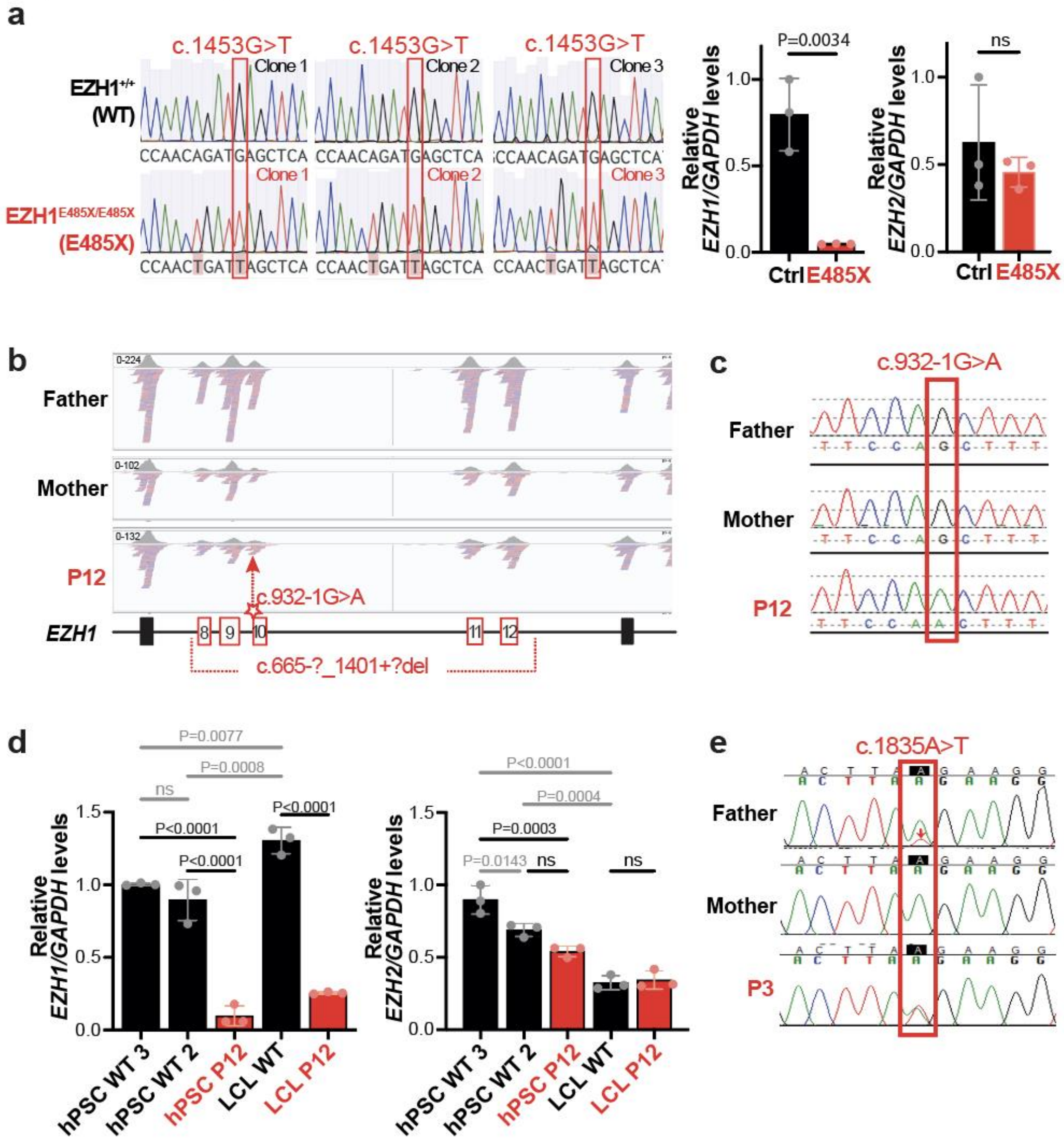

Supplementary Figure 2: Chromatograms and qPCRs of cells carrying *EZH1* variants. a, Sanger sequencing of three hPSC clones carrying the EZH1 p.E485X variant in homozygosity (EZH1$^{E485/E485}$), and their isogenic controls (EZH1$^{+/+}$) generated by CRISPR/Cas9 editing (left). Graphs show mean +/-SD of relative *EZH1/GAPDH* and *EZH2/GAPDH* levels obtained from RT-qPCR of n=3 independent clonal hPSC lines, which highlight the significant reduction of *EZH1* levels and intact *EZH2* in EZH1$^{E485/E485}$ hPSCs compared to isogenic controls. ns=non-significant. Two-sided unpaired t-test. *(right)*. ***b,*** IGV screenshot depicting exon sequencing reads corresponding to *EZH1* exon 7-13 in the genomic DNA of father, mother and the only affected individual (P12) of a non-consanguineous family. The mother and the affected child (P12) show half number of reads covering exon 8-12, which suggests a monoallelic deletion of this region in the mother that was transmitted to the affected child. The arrow points to the de novo splice variant in the affected child (P12). **c,** Sanger sequencing of the genomic DNA extracted from the father, mother, and P12 showing the splice c.932-1G>A variant only in the affected child, thus suggesting a *de novo* origin. Note that the variant falls within the region deleted in the alternate allele. **d,** Graphs show mean +/-SD of relative *EZH1/GAPDH* and *EZH2/GAPDH* levels obtained from RT-qPCR of n=3 independent cell cultures,

which highlight the significant reduction of *EZH1* levels in P12 hPSCs and lymphoblastoid cells (LCL) compared to unrelated wild type (WT) controls. *EZH2* levels are variable between hPSC and LCL, and P12 and control lines. ns=non-significant. One-way ANOVA with Tukey's post hoc analysis test for multiple comparisons. **e,** Sanger sequencing of genomic DNA extracted from the father, mother and affected child (P3) showing the c.1835A>T variant in heterozygosity in P3 and detected at low frequency in the father, who is likely mosaic for the variant. Source data are provided as a Source Data file.

Supplementary Figure 3

a

	p.R406	p.E438	p.K612	p.A678	p.Q731	p.R728	p.L735
D. rerio EZH2	NSRCQ	GAEAS	GAKKH	VDATR	YSQAD	DYRYS	DALKY
G. gallus EZH2	NSRCQ	GAEAS	GSKKH	VDATR	YSQAD	DYRYS	DALKY
H. sapiens EZH2	NSRCQ	GAEAS	GSKKH	VDATR	YSQAD	DYRYS	DALKY
M. musculus EZH2	NSRCQ	GAEAS	GSKKH	VDATR	YSQAD	DYRYS	DALKY
D. rerio EZH1	NSRCP	GAEES	GLKKH	VDATR	YSQAD	DYRYS	DALKY
G. gallus EZH1	NSRCQ	GAEES	GLKKH	VDATR	YSQAD	DYRYS	DALKY
H. sapiens EZH1	NSRCQ	GAEES	GLKKH	VDATR	YSQAD	DYRYS	DALKY
M. musculus EZH1	NSRCQ	GAEES	GLKKH	VDATR	YSQAD	DYRYS	DALKY

b

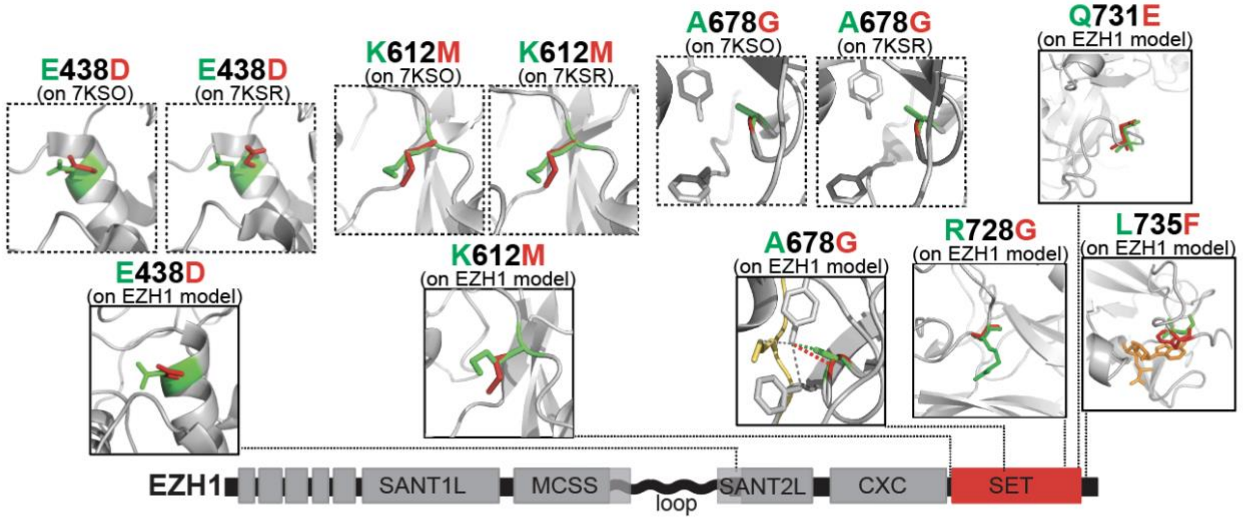

Supplementary Figure 3: Missense *EZH1* variants affect conserved residues and likely impact the interaction of EZH1 with H3K27, SAM or nucleosomes. a, Alignment of human EZH1 and EZH2 protein sequences and their orthologs show that missense variants identified in affected individuals in this study affect residues conserved from zebrafish to humans. **b,** Molecular modeling of missense *EZH1* variants (red) overlapped with the native residue (green) on the experimental 3D structure of EZH1 bound (7KSO) and unbound (7KSR) to nucleosomes for E438D, K612M and A678G or on the computationally predicted EZH1 structure modeled using the experimental structure of EZH2 (PDB: 5HYN) as a template for E438D, K612M, A678G, R728G, Q731E, and L735F variants. The yellow structure in A678G image (on EZH1 model) represents the K27 in the histone tail. The orange structure in the L735F image represents the methyl donor molecule SAM. Note that R406 falls within gaps in all EZH1 structures and R728, Q731 and L735 fall within gaps in the EZH1 experimental 3D structures (7KSO and 7KSR).

Supplementary Figure 4

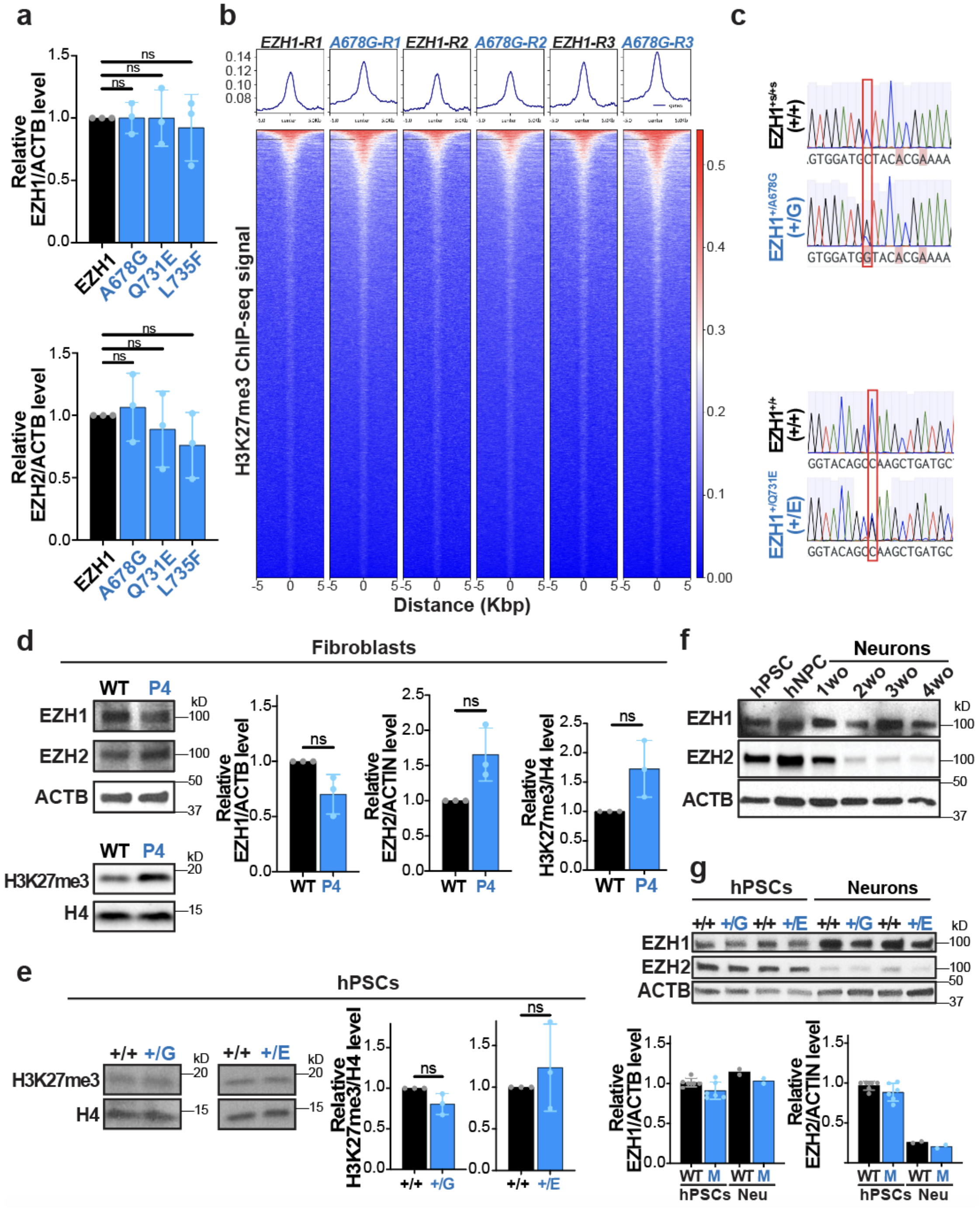

Supplementary Figure 4: *EZH1* missense variants do not affect *EZH1* or *EZH2* expression. a, Graphs showing mean +/-SD of relative EZH1/ACTB and EZH2/ACTB levels quantified by WB band densitometry of n=3 independent ReNcell transductions with wildtype *EZH1* or indicated variants. ns=non-significant. One-way ANOVA with Dunnett's post hoc analysis test for multiple comparisons. **b,** Enrichment plots showing average signal of H3K27me3 in ChIPseq peaks (top) and heatmaps showing normalized H3K27me3 ChIPseq intensities (bottom) ±5kb around the center of the peak in the three replicates of EZH1 or A678G overexpressing ReNcells shown combined in Fig 3c. Each replicate is derived from an independent transduction of ReNcells. **c,** Sanger sequencing of hPSCs edited with CRISPR/Cas9 to carry the indicated missense *EZH1* variants in heterozygosity. The isogenic control for EZH1$^{+/A678G}$ carry two

synonymous variants in homozygosity (EZH1$^{+S/+S}$) introduced during CRISPR/Cas9 editing, whereas the control for EZH1$^{+/Q731E}$ is an unedited clone isolated during the editing process. **d,** WB analysis of EZH1, EZH2 and H3K27me3 in P4 and unrelated control fibroblast lysates. ACTB or H4 are shown as loading controls. Graphs show mean +/-SD of relative EZH1/ACTB, EZH2/ACTB and H3K27me3/H4 levels quantified by WB band densitometry in n=3 independent cell cultures. ns=non-significant. Two-sided paired t-test. **e,** WB analysis of H3K27me3 in hPSCs carrying heterozygous A678G (+/G) or Q731E (+/E) variants in heterozygosity and their isogenic controls. H4 is shown as loading control. Graphs show mean +/-SD of relative H3K27me3/H4 levels quantified by WB band densitometry in n=3 independent cultures. ns=non-significant. Two-sided paired t-test. **f,** WB analysis of EZH1 and EZH2 levels during hPSC to neuronal differentiation, showing a decrease of EZH2 expression beginning at 2 weeks of neuronal differentiation from NPCs. ACTB is shown as loading control. **g,** WB analysis of EZH1 and EZH2 in hPSC and 1-month-old neurons carrying heterozygous A678G (+/G) or Q731E (+/E) variants (M) and their isogenic controls (WT). ACTB is shown as loading control. Graphs show mean of relative EZH1/ACTB and EZH2/ACTB levels quantified by WB band densitometry in n=6 WT and M hPSC and n=2 WT and M independent neuronal cultures. Statistical comparisons are not shown due to small sample size. Source data are provided as a Source Data file.

Supplementary Figure 5

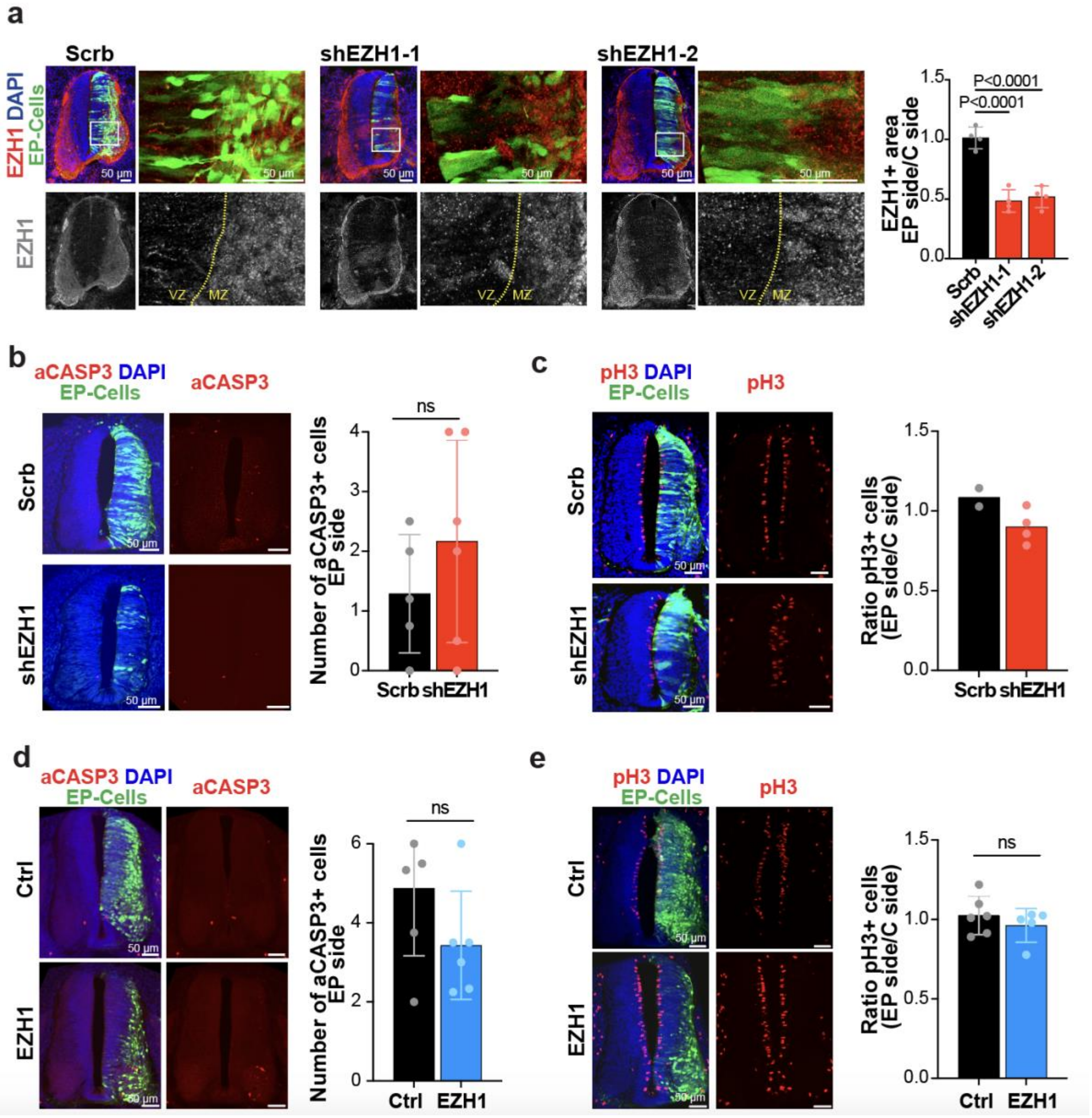

Supplementary Figure 5: Electroporation of shEZH1 reduces EZH1 in the neural tube without affecting survival and mitosis of neural cells. a, Images of EZH1 immunostained neural tubes electroporated with a DNA plasmid encoding EGFP and a scramble shRNA (Scrb) or two different *EZH1* shRNAs (shEZH1) 48h before. Graph show mean +/-SD of the ratio of EZH1 stained area between the electroporated (EP side) and non-electroporated sides (C side) of the neural tubes from n=4 embryos. One-way ANOVA with Dunnett's post hoc analysis test for multiple comparisons. **b,** Images of active Caspase 3 (aCASP3) immunostained transverse neural tube sections 48h after electroporation with Scrb or shEZH1. Graph shows mean +/-SD of n=5 Scrb and n=6 shEZH1 embryos. ns=non-significant. Two-sided Man-Whitney U test. **c,** Images of phosphorylated Histone H3 (pH3) mitosis marker in transverse neural tube sections, 48h after electroporation with Scrb or shEZH1. Graph shows mean n=2 Scrb and n=4 shEZH1 embryos. Statistical comparisons are not shown due to small sample size. **d, e,** Representative image aCASP3 (d) and pH3 mitosis marker (e) in transverse neural tube sections 48h after electroporation with control or EZH1 encoding plasmids. Graphs show mean +/-SD of n=5 Ctrl, n=6 EZH1 (d), and n=6 Ctrl and

n=5 EZH1 (e) embryos. ns=non-significant. Two-sided Man-Whitney U test. Source data are provided as a Source Data file.

Supplementary Figure 6

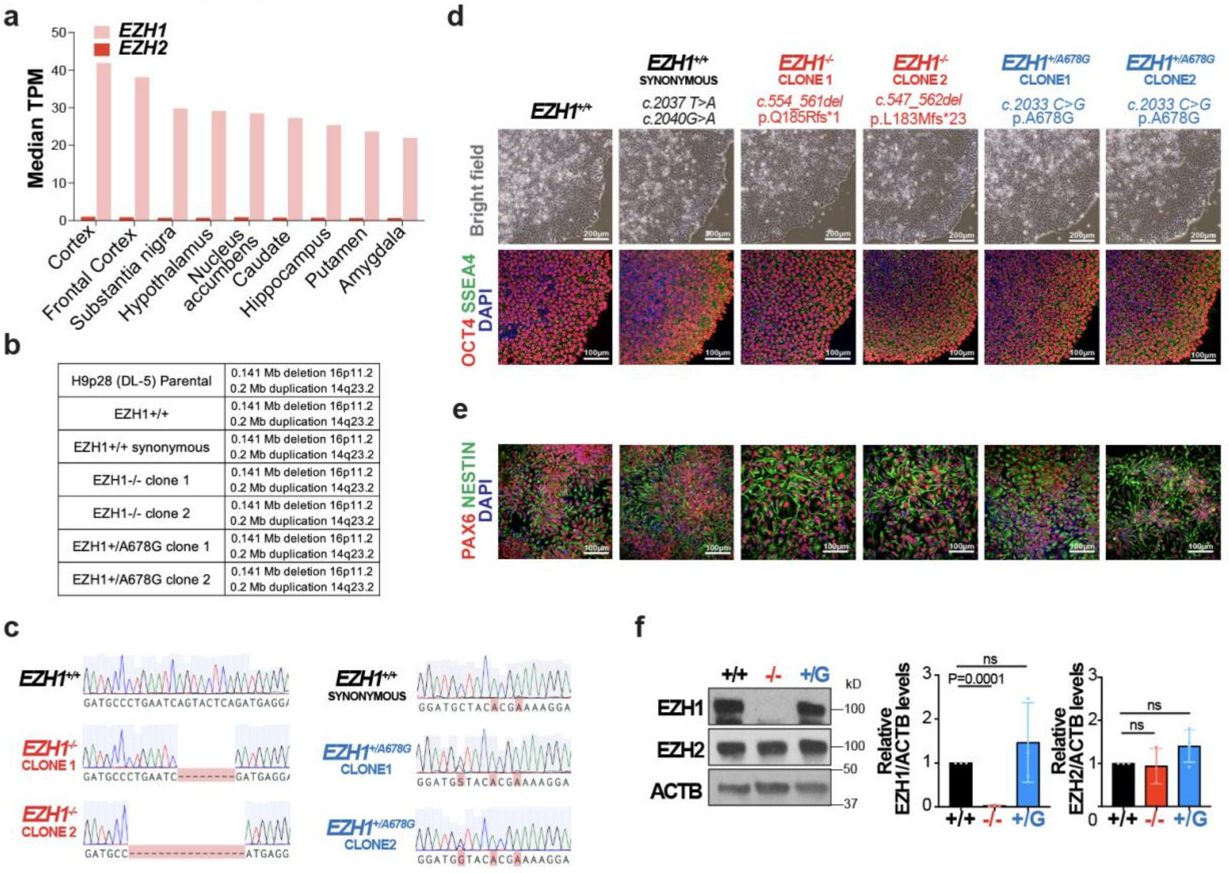

Supplementary Figure 6: Human brain *EZH1* expression and validation of hPSC editing and differentiation. a, *EZH1* and *EZH2* expression levels (median TPM) on the indicated regions of the adult human brains. Data mined from mRNA-sequencing datasets in GTEX. **b,** Results from CNV analysis of the parental H9 hPSCs, non-edited H9 clone, H9 edited to carry *EZH1* synonymous variants, two clones carrying *EZH1* LOF variant ($EZH1^{-/-}$) and one of the patients' GOF variants ($EZH1^{+/A678G}$). **c,** Sanger sequencing chromatograms showing *EZH1* variants in edited hPCS clones. **d,** Bright field images and immunostainings for pluripotency markers OCT4 and SSEA4 of hPSCs carrying *EZH1* LOF or GOF variants introduced by CRIPSR/Cas9 genome editing representative of at least two independently immunostained cultures. **e,** Images of hPSC-derived neural progenitor cells immunostained for PAX6 and NESTIN (NPC markers) representative of at least two independently immunostained cultures. **f,** Western blot analysis of EZH1 and EZH2 in hPSCs showing undetectable levels of EZH1 in hPSCs carrying a homozygous loss of function variant ($EZH1^{-/-}$) and intact levels in hPSCs carrying a gain of function variant ($EZH1^{+/A678G}$). EZH2 levels are similar between genotypes. ACTB is shown as loading control. Graphs show mean +/-SD of n=3 independent cultures. ns=non-significant. One-way ANOVA with Dunnett's post hoc analysis test for multiple comparisons. Source data are provided as a Source Data file.

Supplementary Figure 7

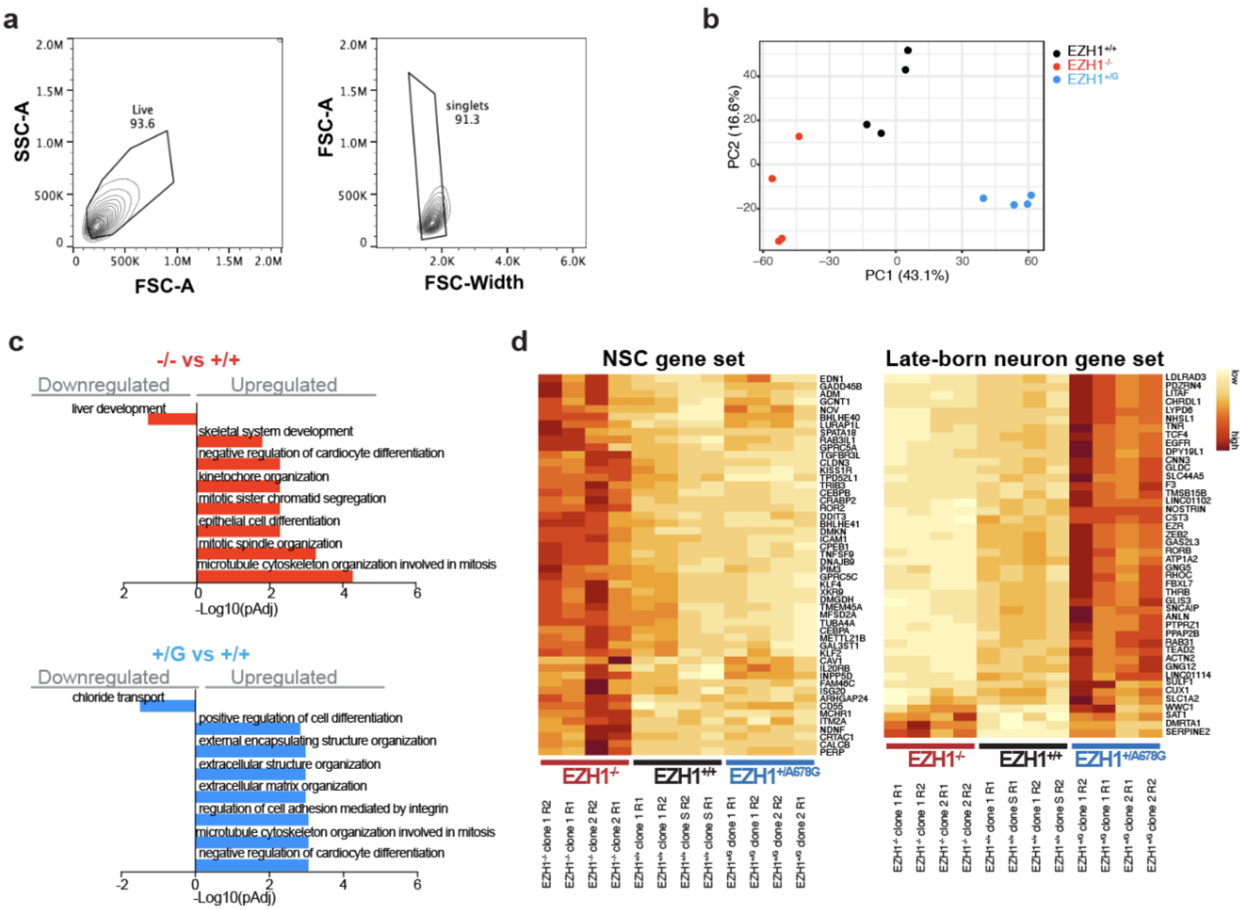

Supplementary Figure 7: Flow cytometry gating and hPSC RNAseq analysis. a. Plots illustrating the gating strategy used for Fig 5c and d flow cytometry analysis. The starting live cells were selected using Forward Side Scatter-Area (FSC-A) and Side Scatter-Area (SSC-A) and duplets were excluded by selecting singlet cell population within the indicated FSC-A and FSC-Width coordinates. **b,** PCA plot of RNAseq analysis performed on EZH1$^{+/+}$ (+/+), EZH1$^{-/-}$ (-/-) and EZH1$^{+/A678G}$ (+/G) 2-month-old neurons. Two clones per genotype, each differentiated twice (for a total of 4 replicates) were analyzed. **c,** Gene ontology analysis for Biological Process of genes differentially expressed in -/- or +/G compared to +/+. Adjusted p-values (pAdj) were computed from the Fisher exact test with Benjamini-Hochberg adjustment for multiple comparison. Differentially expressed genes were defined as those with absolute $\log_2$(FoldChange>1) and padj<0.05 with the Wald test with Benjamini-Hochberg method for multiple comparison. **d,** Heatmap showing the expression level (read counts) of genes contributing to the leading edge of neural stem cell (NSC) gene set in -/- vs +/+ and the leading edge of late-born neuron gene set in +/G vs +/+ in GSEA analysis.

Supplementary Figure 8

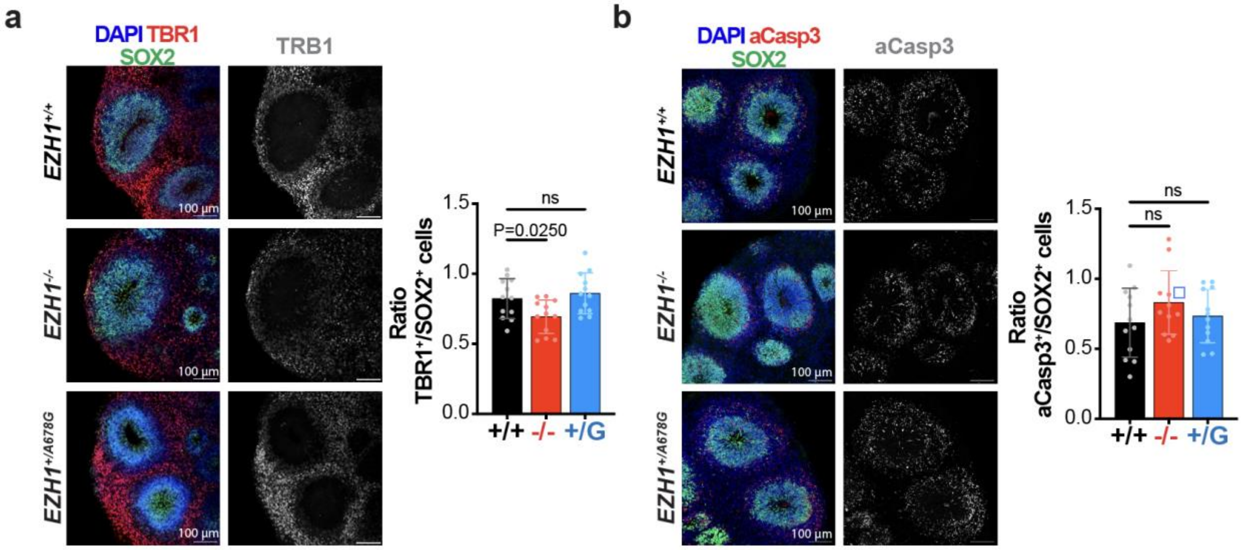

Supplementary Figure 8: Forebrain organoids derived from EZH1 LOF show slightly reduced TBR1^{+} deep layer neuron population and normal levels of cell death.

a, Images of 60-day old forebrain cortical organoid sections immunostained for TBR1 (layer VI early born neuron marker) and SOX2 (neural progenitor marker). Graph shows mean +/-SD of the number of TBR1^{+} cells over SOX2^{+} cells in n=12 organoids. ns=non-significant. Two-sided unpaired t-test with Holm-Sidak post hoc analysis test for multiple comparisons. **b,** Representative images of 60-day old forebrain cortical organoid sections immunostained for aCasp3 and SOX2. Graph shows mean +/-SD of the number of aCasp3^{+} cells over SOX2^{+} cells for n=12 organoids from two independent batches. ns=non-significant. Two-sided unpaired t-test. Source data are provided as a Source Data file.

Supplementary Table 1: Variant features

Patient ID	P1	P2	P3	P4	P5	P6	P7	P8	P9	P10	P11	P12	P13	P14	P15	P16	P17	P18	P19
**Method of Identifying Variant**	Exome Sequencing	Exome Sequencing	Exome Sequencing	Trio Exome Sequencing and Quad genome sequencing (including unaffected sibling)	Exome Sequencing	Exome Sequencing	Exome Sequencing	Exome Sequencing	Exome based panel test	Exome Sequencing	Exome Sequencing	Trio Exome Sequencing	Sanger Sequencing	Sanger Sequencing	Exome Sequencing	Sanger Sequencing	Solo Exome Sequencing	Exome Sequencing	Exome Sequencing
**Possition in genome (GRCh37-v1.6)**	Chr17:40864491 C>T	chr17:40864394 T>G	Chr17:40858029 T>A	Chr17:40855823 G>C	Chr17:40854896 T>C	Chr17:40854896 T>C	Chr17:40854603 G>C	Chr17:40854591 G>A	Chr17:40854591 G>A	Chr17:4087063 1 G>A	Chr17:4087063 1 G>A	Chr17:40864307-?_40871225+?del and Chr17:40870086 C>T	chr17:40861904 C>A	chr17:40861904 C>A	chr17:40861904 C>A	chr17:40861904 C>A	chr17:40861904 C>A	chr17:40861904 C>A	chr17:40864471 G>A
**Coding Sequence Change (NM_001991.3)**	c.1217G>A	c.1314A>C	c.1835A>T	c.2033C>G	c.2182A>G	c.2182A>G	c.2191C>G	c.2203C>T	c.2203C>T	c.772C>T	c.772C>T	c.[664+1_665-1]_[1401+1_1402-1]del and c.932-1G>A	c.1453G>T	c.1453G>T	c.1453G>T	c.1453G>T	c.1453G>T	c.1453G>T	c.1137C>T
**Protein Sequence Change**	p.R406H	p.E438D	p.K612M	p.A678G	p.R728G	p.R728G	p.Q731E	p.L735F	p.L735F	p.R258X	p.R258X	p. ?	p.E485X	p.E485X	p.E485X	p.E485X	p.E485X	p.E485X	p.Q413X
**dN/dS**	0.12	0.5	0.28	0.06	0.17	0.17	0.25	0.26	0.26										
**Exon Number Position**	Exon 12	Exon 12	Exon 16	Exon 19	Exon 20	Exon 20	Exon 21	Exon 21	Exon 21	Exon 9	Exon 9	Exon 8-12	Exon 13	Exon 13	Exon 13	Exon 13	Exon 13	Exon 13	Exon 12
**Codon Change**	CGC>CAC	GAA>GAC	AAG>ATG	GCT>GGT	AGG>GGG	AGG>GGG	CAA>GAA	CTC>TTC	CTC>TTC	TCG>TAG	TCG>TAG		GAG>TAG	GAG>TAG	GAG>TAG	GAG>TAG	GAG>TAG	GAG>TAG	CAG>TAG
**Consequence**	Aminoacid change	Aminoacid change	Aminoacid change	Aminoacid change	Aminoacid change	Aminoacid change	Aminoacid change	Aminoacid change	Aminoacid change	Stop gain	Stop gain	Deletio and splicing error	Stop gain	Stop gain	Stop gain	Stop gain	Stop gain	Stop gain	Stop gain
**Zygosity**	Heterozygous	Heterozygous	Heterozygous	Heterozygous	Heterozygous	Heterozygous	Heterozygous	Heterozygous	Heterozygous	Homozygous	Homozygous	Compound Heterozygous	Homozygous	Homozygous	Homozygous	Homozygous	Homozygous	Homozygous	Homozygous
**Missense Tolerance Ratio (Metadome)**	0.12 Highly Intolerant	0.5 Intolerant	0.28 Intolerant	0.06 Highly Intolerant	0.17 Highly Intolerant	0.17 Highly Intolerant	0.25 Intolerant	0.26 Intolerant	0.26 Intolerant										
**Polyphen**	0.980 Probably damaging	0.007 Benign	1.000 Probably damaging	1.000 Probably Damaging	1.000 Probably Damaging	1.000 Probably Damaging	0.916 Possibly damaging	0.997 Probably Damaging	0.997 Probably Damaging										
**MutationTaster**	29 (disease causing)	45 (disease causing)	95 (disease causing)	60 (disease causing)	125 (disease causing)	125 (disease causing)	29 (disease causing)	22 (disease causing)	22 (disease causing)	6 (disease causing)	6 (disease causing)		6 (disease causing)	6 (disease causing)	6 (disease causing)	6 (disease causing)	6 (disease causing)	6 (disease causing)	6 (disease causing)
**CADD score**	29.9	18.45	30	32	33	33	22.7	31	31	35	35		39	39	39	39	39	39	37
**Variant frequency**	Not found in ExaC, GnomAD or 1000g, seen in GeneDX database in presumably healthy parents	Not found in ExaC, GnomAD or 1000g	Not found in ExaC, GnomAD or 1000g	Not found in ExaC, GnomAD or 1000g	Not found in ExaC, GnomAD or 1000g	Not found in ExaC, GnomAD or 1000g	Not found in ExaC, GnomAD or 1000g	Not found in ExaC, GnomAD or 1000g, seen in GeneDX database in presumably healthy parents	Not found in ExaC, GnomAD or 1000g, seen in GeneDX database in presumably healthy parents	Not found in 1000G, 1 carrier found in ExaC	Not found in 1000G, 1 carrier found in ExaC	Not found in ExaC, GnomAD or 1000g	Not found in ExaC, GnomAD or 1000g	Not found in ExaC, GnomAD or 1000g	Not found in ExaC, GnomAD or 1000g	Not found in ExaC, GnomAD or 1000g	Not found in ExaC, GnomAD or 1000g	Not found in ExaC, GnomAD or 1000g	Not found in ExaC, GnomAD or 1000g

Supplementary Table 2: RT and PCR primers

Primer Name	Sequence (5'->3')	Note
Targeted RT-Splice EX20-21	GCTTGGCTGTACCTGTAATCAAAG	For targeted retrotranscription: targets 5' end of exon 21_3' end of exon 20
Targeted RT-GAPDH EX8-9	CGTTGTCATACCAGGAAATGAGC	For targeted retrotranscription: targets 5' end of exon 9_3' end of exon 8
RTPCR Splice Exon 9 FW	CCTCAGTGCACACCCAACA	For RT-PCR Exon 9
RTPCR Splice Exon 10 RV	GCAGTCTGTGCCACATGGTTCTGG	For RT-PCR Exon 10
RTPCR Splice Exon 11 RV	GCCCAGTCATTGCCTGTGTCCC	For RT-PCR Exon 11
RTPCR GAPDH FW	GGATTTGGTCGTATTGGG	For RT-PCR Exon 2
RTPCR GAPDH RV	GGAAGATGGTGATGGGATT	For RT-PCR Exon 3
qRTPCR hEZH1 3' FW	GTAGTGGATGCTACTCGGAAAG	For qRTPCR Targets 3' end
qRTPCR hEZH1 3' RV	AGCTTGGCTGTACCTGTAATC	For qRTPCR Targets 3' end
qRTPCR hGAPDH FW	CCATGGGGAAGGTGAAGGTC	For qRTPCR Exon 2
qRTPCR hGAPDH RV	TGGAATTTGCCATGGGTGGA	For qRTPCR Exon 4

Supplementary Table 3: Oligonucleotides used for hPSC editing and testing

Cell line (genotype)	Oligonucleotide	sequence	targeted exon
EZH1-/-	sgRNA	cctgaatcagtactcagatg	Exon 7
	sequencing primer F	accactgctcatctttcgaatc	
	sequencing primer R	acgtaccttcaatagcatgtcg	
EZH1+/A678G	sgRNA	gtagtggatgctactcggaa	Exon 19
	ssODN_patient mutation and silent mutations	ctgtgcttatggttttccttgatttactttatacagattttgtagtggatgct acacgaaaaggaaacaaaattcgatttgcaaatcattcagtgaat cccaac	
	ssODN_silent mutations only	ctgtgcttatggttttccttgatttactttatacagattttgtagtggatggt acacgaaaaggaaacaaaattcgatttgcaaatcattcagtgaat cccaac	
	sequencing primer F	ctgagaagctgtggaattgc	
	sequencing primer R	tcacagtaaagaggcatccatc	
EZH1+/Q731E	sgRNA	cgtacttgagagcatcagct	Exon 21
	ssODN_patient mutation and silent mutations	agtcatgagagtaacctggttcctcttccctgctctgggacaggtaca gcgaagctgatgctctcaagtacgtggggatcgagagggagacc gacgtccttta	
	sequencing primer F	tttgtgccctctggacatgg	
	sequencing primer R	tagatttgggggctcaggga	
EZH1 E485X/E485X	sgRNA	gggttcatgagctcatctgt	Exon 13
	ssODN_patient mutation and silent mutations	tctttcagtttgcagtcaaagaatcacttatcctgaagctgccaactg attagctcatgaacccctcacagaagaagaaaagaaagcacag gcaagaaggg	
	sequencing primer F	cacccagctgggccacttac	
	sequencing primer R	ggcagatgggaactccaaggg	
