# Supplementary material for: Gain and loss of function variants in EZH1 disrupt neurogenesis and cause dominant and recessive neurodevelopmental disorders
Source: Nat Commun. 2023 Jul 11;14:4109. doi: 10.1038/s41467-023-39645-5 (PMC10336078; doi:10.1038/s41467-023-39645-5)
Supplement: Supplementary file 3 — Description of Additional Supplementary Files [file 41467_2023_39645_MOESM3_ESM.pdf]

File Name: Supplementary Data 1
Description: Clinical findings in individuals with EZH1 mutations. NR=Not Reported. NICU=Neonatal Intensive Care Unit. IVH=Intraventricular Hemorrhage. OCD=Obsessive Compulsive Disorder. CT=Computerized tomography. VLP=Variant of Low Pathogenic score. EEG=Electroencephalogram

File Name: Supplementary Data 2
Description: Differentially expressed genes in EZH1-/- vs EZH1+/+ 2-month-old hPSC derived neurons.

File Name: Supplementary Data 3
Description: Differentially expressed genes in EZH1+/G vs EZH1+/+ 2-month-old hPSC derived neurons.
