# Supplementary material for: Gain and loss of function variants in EZH1 disrupt neurogenesis and cause dominant and recessive neurodevelopmental disorders
Source: Nat Commun. 2023 Jul 11;14:4109. doi: 10.1038/s41467-023-39645-5 (PMC10336078; doi:10.1038/s41467-023-39645-5)
Supplement: Supplementary file 7 — Reporting Summary [file 41467_2023_39645_MOESM7_ESM.pdf]

nature portfolio

Corresponding author(s): Naiara Akizu

Last updated by author(s): 05/04/2023

Reporting Summary

Nature Portfolio wishes to improve the reproducibility of the work that we publish. This form provides structure for consistency and transparency in reporting. For further information on Nature Portfolio policies, see our Editorial Policies and the Editorial Policy Checklist.

Statistics

For all statistical analyses, confirm that the following items are present in the figure legend, table legend, main text, or Methods section.

n/a	Confirmed	
☐	☒	The exact sample size (*n*) for each experimental group/condition, given as a discrete number and unit of measurement
☐	☒	A statement on whether measurements were taken from distinct samples or whether the same sample was measured repeatedly
☐	☒	The statistical test(s) used AND whether they are one- or two-sided<br>*Only common tests should be described solely by name; describe more complex techniques in the Methods section.*
☐	☒	A description of all covariates tested
☐	☒	A description of any assumptions or corrections, such as tests of normality and adjustment for multiple comparisons
☐	☒	A full description of the statistical parameters including central tendency (e.g. means) or other basic estimates (e.g. regression coefficient) AND variation (e.g. standard deviation) or associated estimates of uncertainty (e.g. confidence intervals)
☐	☒	For null hypothesis testing, the test statistic (e.g. *F*, *t*, *r*) with confidence intervals, effect sizes, degrees of freedom and *P* value noted<br>*Give P values as exact values whenever suitable.*
☒	☐	For Bayesian analysis, information on the choice of priors and Markov chain Monte Carlo settings
☒	☐	For hierarchical and complex designs, identification of the appropriate level for tests and full reporting of outcomes
☒	☐	Estimates of effect sizes (e.g. Cohen's *d*, Pearson's *r*), indicating how they were calculated

Our web collection on statistics for biologists contains articles on many of the points above.

Software and code

Policy information about availability of computer code

Data collection	Immunostaining data collection was performed using Leica SP8, Zeiss LSM 780, and Zeiss LSM 800. BD FACSAria™ Fusion Flow Cytometer for fluorescent cell sorting and CytoFLEX (Beckman Coulter) for flow cytometry analysis. Gels were imaged using BioRad Gel Doc EZ Imager. RT-qPCR were performed using Biorad CFX384 Touch Real-Time PCR. Western Blot films were developed using the AFP Mini-Med 90 X-Ray Film Processor.
Data analysis	Immunistaining and Western blot image processing, analysis and quantification was done with ImageJ software (Fiji, version 2.0.20-rc-69/1.5n, U. S. National Institutes of Health, Bethesda, Maryland, USA, https://imagej.nih.gov/ij/, 1997-2018.) and Photoshop (version 23.0.1). For data statistical analysis and plotting Excel (Microsoft Version 16.72), GraphPad (Prism Version 9.1.0, GraphPad Software, LLC) and ggplot (version 3.4.2) were used. Flow cytometry data analysis was perfomed on the FlowJo (version 10.8.2). FACS analysis was performed using BD FACSDiva (version 9.0.1). For RNAseq analysis, STAR (version 2.7.1a), featureCounts (subread-1.6.1), DEseq2 (v1.28.1) and ClusterProfiler (v.4.0.5) were used. For ChIPseq analysis, bowtie2 (v2.1.0), samtools (v0.1.19), MACS2 (v2.2.7.1) and deepTools (v3.5.0) were used. For protein sequence and structure analysis ClustalW2 and Modeller were used. For exome sequencing BWA (v0.7.1276), Picard (v1.97), GATK (v2.6.577), ANNOVAR and SnpEff were used.

For manuscripts utilizing custom algorithms or software that are central to the research but not yet described in published literature, software must be made available to editors and reviewers. We strongly encourage code deposition in a community repository (e.g. GitHub). See the Nature Portfolio guidelines for submitting code & software for further information.

Data

Policy information about availability of data

All manuscripts must include a data availability statement. This statement should provide the following information, where applicable:

- Accession codes, unique identifiers, or web links for publicly available datasets
- A description of any restrictions on data availability
- For clinical datasets or third party data, please ensure that the statement adheres to our policy

All data supporting the findings of this study are available within the article and its supplementary information files. Source data are provided with this paper. The exome/genome sequencing will be made available upon request provided that privacy and consent criteria are preserved. The ChIPseq and RNAseq data generated in this study have been deposited in GEO under accession code GSE210465 [https://www.ncbi.nlm.nih.gov/geo/query/acc.cgi?acc=GSE210465] and GSE227014 [https://www.ncbi.nlm.nih.gov/geo/query/acc.cgi?acc=GSE227014]. Other raw data will be made available within two weeks upon request to corresponding author. The human brain gene expression data used in this study are available in BrainSpan (https://www.brainspan.org/rnaseq/searches?exact_match=false&search_term=EZH1&search_type=gene and https://www.brainspan.org/rnaseq/searches?exact_match=false&search_term=EZH2&search_type=gene&page_num=0) and GTEX (https://gtexportal.org/home/gene/EZH1 and https://gtexportal.org/home/gene/EZH2). Variants p.R406H and p.A678G are accessible in ClinVar under the accession codes VCV000828189.1 and VCV000977755.2 respectively. The other variants characterized in this study have been submitted to ClinVar.

Human research participants

Policy information about studies involving human research participants and Sex and Gender in Research.

Reporting on sex and gender	Since this study is focused on gene identification, all patients with confirmed diagnosis are included in the study, regardless of their gender. Gender of each patient is indicated in Supplementary Table 1.
Population characteristics	Patients included in the study were identified based on genetic diagnostic through national and international collaborations and belong to diverse origins, including Middle East, Europe and USA (see Supplementary Table 1).
Recruitment	Patients were recruited through collaborations with local and international clinicians and geneticists. These connections were facilitated by Genematcher, Deciphering Developmental Disorders project, and 100,000 Genomes Project.
Ethics oversight	This study was approved by the Institutional Review Boards of the Children's Hospital of Philadelphia, Boston Children's Hospital, University College London, Guy's Hospital, Kennedy Krieger Institute, King Faisal Specialist Hospital & Research Center and University of Alabama in Birmingham. Ethical approval and informed consent were obtained for participation, phenotyping, sample collection and generation/derivation of patient and control cell lines including fibroblasts, lymphoblastoid cell lines, peripheral blood mononuclear cells and their reprograming. The authors also confirm that human research participants provided written informed consent for publication of the images in Figure 1 and Supplementary Figure 1.

Note that full information on the approval of the study protocol must also be provided in the manuscript.

Field-specific reporting

Please select the one below that is the best fit for your research. If you are not sure, read the appropriate sections before making your selection.

☒ Life sciences ☐ Behavioural & social sciences ☐ Ecological, evolutionary & environmental sciences

For a reference copy of the document with all sections, see nature.com/documents/nr-reporting-summary-flat.pdf

Life sciences study design

All studies must disclose on these points even when the disclosure is negative.

Sample size	Detailed sample size information was labeled with individual dots in each graph and noted in the figure legends. Specifically, RT-qPCR quantification was averaged from 3 technical repeats. Western blot was analyzed with 2-7 independent cell lysates derived from independently cultured or independently generated cells lines (for lentiviral transductions and hPSC clones). ChIP-seq data is shown as merged from 3 independent biological replicates (in Fig 3c) and individually for each replicate (in Supplementary Fig 4b). In vitro histone methyltransferase assays were performed 2-3 times independently, with two concentration tested in each time. For the chick embryo electroporation experiments, 2-8 electroporated embryos were analyzed per experiment (for each embryo data represents the average of the quantifications from 1-5 sections). For hPSC and hNPC experiments, samples were collected from 3 independent differentiations. For organoid experiments of each time point, data from 2 independent differentiations with 11-23 organoids per batch were collected to quantify VZ thickness, and data from 2 independent differentiations with 5-7 organoids per batch were collected to quantify neuronal identity. The sample size and statistical tests were chosen based on previous studies with similar methodologies (Saade et al., 2017, Nat Cell Biol; Akizu et al., 2013, Cell; Qian et al., 2020, Cell Stem Cell) and the data met the assumptions for each statistical test performed.

Data exclusions	No data was excluded from the analyses.
Replication	To ensure reproducibility, each experiment was successfully reproduced at least twice independently.
Randomization	The data were grouped by genotypes. The collecting and processing were randomly performed.
Blinding	Blinding was not performed since no data exclusions were done and all measurements were taken objectively.

Reporting for specific materials, systems and methods

We require information from authors about some types of materials, experimental systems and methods used in many studies. Here, indicate whether each material, system or method listed is relevant to your study. If you are not sure if a list item applies to your research, read the appropriate section before selecting a response.

Materials & experimental systems

n/a	Involved in the study	
☐	☒	Antibodies
☐	☒	Eukaryotic cell lines
☒	☐	Palaeontology and archaeology
☐	☒	Animals and other organisms
☒	☐	Clinical data
☒	☐	Dual use research of concern

Methods

n/a	Involved in the study	
☐	☒	ChIP-seq
☐	☒	Flow cytometry
☒	☐	MRI-based neuroimaging

Antibodies

Antibodies used

ChIP-seq
antiH3K27me3 EMD (Millipore 07-449)

Western Blots
antiEZH1 (in-house Reinberg lab antibody; Margueron et al., 2008 Mol Cell)
antiEZH1 (Proteintech, #20852-1AP)
antiEZH2 (Cell Signaling D2C9 #5246)
antiH3K27me3 (EMD Millipore, 07-449)
antiβ-actin (GenScript A00702)
antiH4 (Abcam ab10158)
antiRabbit HRP (Invitrogen, #31458)
antiMouse HRP (Invitrogen, #SA1-100)

hPSCs and derived neural cells Immunostainings
antiSSEA (Abcam ab16287)
antiOCT4 (Abcam ab19857)
antiPAX6 (Biolegend 901302)
antiNestin (Millipore Sigma MAB5326)
antiTUJ1 (Abcam ab18207)

Chick-embryo Immunostainings
antiHuC/D (Millipore A-21271)
antiNeuN, clone A60 (Chemicon MAB377)
antiSOX2 (Abcam AB97959)
antiSOX9 (in-house James Briscoe lab- Cheung M, et al. Dev Cell 8(2):179-192)

Cortical Organoid Immunostainings
antiSOX2 (R&D Systems AF2018)
antiCTIP2 (Abcam ab18465)
antiSATB2 (Abcam ab51502)
antiTBR1 (Abcam ab31940)
aCasp3 (Cell Signaling, #9661)

Secondary antibodies for Immunofluorescences
Anti-rabbit Alexa Fluor 488 (Invitrogen A11008)
Anti-rabbit Alexa Fluor 555 (Invitrogen A21434)
Anti-rabbit Alexa Fluor 633 (Invitrogen A21070)

Anti-mouse Alexa Fluor 488 (Invitrogen A11001)
Anti-mouse Alexa Fluor 555 (Invitrogen A21422)
Anti-mouse Alexa Fluor 633 (Invitrogen A21050)
Anti-rabbit Alexa Fluor 488 (Invitrogen A21206)
Anti-rat Alexa Fluor 647 (Invitrogen A78947)
Anti-mouse Alexa Fluor 555 (Invitrogen A31570)
Anti-mouse IgG Cy5 (Jackson ImmunoResearch #115175146)

Flow Cytometry
anti-Ki67 (Cell Signaling #9449)
anti-HuC/D (Invitrogen A-21271)
anti-SOX2 (Cell Signaling #3579)
goat anti-mouse IgG1-conjugated Alex 647 (Jackson ImmunoResearch 115-605-205)
goat anti-mouse IgG2b-conjugated Alexa 488 (Jackson ImmunoResearch 115-545-207)
goat anti-rabbit conjugated brilliant violet 421 (Jackson ImmunoResearch 111-675-144)

Validation

Antibodies meet all of the quality control standards defined by manufactures. Validation statements for the commercial antibodies are available on the manufactures websites. Custom made antibodies were validated in publications and further validated by us as indicated below:

antiH3K27me3 (Millipore; https://www.sigmaaldrich.com/US/en/product/mm/07449)
antiEZH1 (This antibody has been previously validated (Margueron et al., 2008 Mol Cell). We further knockout-validated the antibody prior to use.)
antiEZH1(https://www.ptglab.com/products/EZH1-Antibody-20852-1-AP.htm)
antiEZH2 (https://www.cellsignal.com/products/primary-antibodies/ezh2-d2c9-xp-rabbit-mab/5246)
antiH3K27me3 (https://www.cellsignal.com/products/primary-antibodies/tri-methyl-histone-h3-lys36-d5a7-xp-rabbit-mab/4909)
antiβ-actin (https://www.genscript.com/antibody/A00702-THE_beta_Actin_Antibody_mAb_Mouse.html)
antiH4 (https://www.abcam.com/histone-h4-antibody-chip-grade-ab10158.html)
antiRabbit HRP (https://www.thermofisher.com/antibody/product/Donkey-anti-Rabbit-IgG-H-L-Cross-Adsorbed-Secondary-Antibody-Polyclonal/31458)
antiMouse HRP (https://www.thermofisher.com/antibody/product/Donkey-anti-Mouse-IgG-H-L-Cross-Adsorbed-Secondary-Antibody-Polyclonal/SA1-100)
antiSSEA (https://www.abcam.com/ssea4-antibody-mc813-70-ab16287.html)
antiOCT4 (https://www.abcam.com/oct4-antibody-ab19857.html)
antiPAX6 (https://www.biolegend.com/fr-fr/products/purified-anti-pax-6-antibody-11511)
antiNestin (https://www.sigmaaldrich.com/US/en/product/mm/mab5326)
antiTUJ1 (https://www.abcam.com/beta-iii-tubulin-antibody-neuronal-marker-ab18207.html)
antiHuC/D (https://www.thermofisher.com/antibody/product/HuC-HuD-Antibody-clone-16A11-Monoclonal/A-21271_)
antiNeuN (https://www.emdmillipore.com/US/en/product/Anti-NeuN-Antibody-clone-A60,MM_NF-MAB377#documentation)
antiSOX2 (R&D Systems; https://www.rndsystems.com/products/human-mouse-rat-sox2-antibody_af2018)
antiSOX2(Abcam; https://www.abcam.com/sox2-antibody-ab97959.html)
antiSOX9 (or validation: Cheung M, et al. Dev Cell 8(2):179-192). We further validated the antibody by cell type specificity in the chick embryo neural tube.
antiCTIP2 (https://www.abcam.com/ctip2-antibody-25b6-ab18465.html)
antiSATB2 (https://www.abcam.com/satb1--satb2-antibody-satba4b10-c-terminal-ab51502.html)
antiTBR1 (https://www.abcam.com/products/primary-antibodies/tbr1-antibody-ab31940.html)
antiCasp3 (https://www.cellsignal.com/products/primary-antibodies/cleaved-caspase-3-asp175-antibody/9661)
antiKi67 (https://www.cellsignal.com/products/primary-antibodies/ki-67-8d5-mouse-mab/9449)
antiHuC/D (https://www.thermofisher.com/antibody/product/HuC-HuD-Antibody-clone-16A11-Monoclonal/A-21271)
antiSOX2 (https://www.cellsignal.com/products/primary-antibodies/sox2-d6d9-xp-rabbit-mab/3579)
goat anti-mouse IgG1-conjugated Alex 647 (https://www.jacksonimmuno.com/catalog/products/115-605-205)
goat anti-mouse IgG2b-conjugated Alexa 488 (https://www.jacksonimmuno.com/catalog/products/115-545-207)
goat anti-rabbit conjugated brilliant violet 421 (https://www.jacksonimmuno.com/catalog/products/111-675-144)
Anti-rabbit Alexa Fluor 488 (https://www.thermofisher.com/antibody/product/Goat-anti-Rabbit-IgG-H-L-Cross-Adsorbed-Secondary-Antibody-Polyclonal/A-11008)
Anti-rabbit Alexa Fluor 555 (https://www.thermofisher.com/antibody/product/Goat-anti-Rabbit-IgG-H-L-Cross-Adsorbed-Secondary-Antibody-Polyclonal/A-21428)
Anti-rabbit Alexa Fluor 633 (https://www.thermofisher.com/antibody/product/Goat-anti-Rabbit-IgG-H-L-Cross-Adsorbed-Secondary-Antibody-Polyclonal/A-21070)
Anti-mouse Alexa Fluor 488 (https://www.thermofisher.com/antibody/product/Goat-anti-Mouse-IgG-H-L-Cross-Adsorbed-Secondary-Antibody-Polyclonal/A-11001)
Anti-mouse Alexa Fluor 555 (https://www.thermofisher.com/antibody/product/Goat-anti-Mouse-IgG-H-L-Cross-Adsorbed-Secondary-Antibody-Polyclonal/A-21422)
Anti-mouse Alexa Fluor 633 (https://www.thermofisher.com/antibody/product/Goat-anti-Mouse-IgG-H-L-Cross-Adsorbed-Secondary-Antibody-Polyclonal/A-21050)
Anti-rabbit Alexa Fluor 488 (https://www.thermofisher.com/antibody/product/Donkey-anti-Rabbit-IgG-H-L-Highly-Cross-Adsorbed-Secondary-Antibody-Polyclonal/A-21206)
Anti-rat Alexa Fluor 647 (https://www.thermofisher.com/antibody/product/Donkey-anti-Rat-IgG-H-L-Highly-Cross-Adsorbed-Secondary-Antibody-Polyclonal/A78947)
Anti-mouse Alexa Fluor 555 (https://www.thermofisher.com/antibody/product/Donkey-anti-Mouse-IgG-H-L-Highly-Cross-Adsorbed-Secondary-Antibody-Polyclonal/A-31570)
Anti-mouse IgG Cy5 (https://www.jacksonimmuno.com/catalog/products/115-175-146)

Eukaryotic cell lines

Policy information about cell lines and Sex and Gender in Research

Cell line source(s)	ReNcell VM are a male, immortalized human neural progenitor cell line (Sigma Aldrich SCC008). H9 hESCs are female human embryonic stem cells (WiCell WA09) and KOLF2.1J hPSCs are human induced pluripotent stem cells of a male of European Ancestry (from the Jackson Laboratory JIPSC1000). iPSCs were generated in the CHOP stem cell core from PBMCs. LCLs were generated from peripheral blood lymphocytes. Fibroblasts were derived from skin biopsies. HEK293-T are immortalized epithelial like cells from a human kidney (ATCC CRL-3216).
Authentication	ReNcell VM identity was confirmed by assessment of morphological features and ability of these cells to differentiate to neuron-like cells. LCL identity was confirmed by their growing capacity in suspension, round morphology and genotyping by Sanger Sequencing of EZH1 variant. Fibroblast identity was confirmed by their elongated morpholgy and genotyping by Sanger Sequencing of EZH1 variant. hESC and iPSCs were obtained from CHOP Stem Cell Core, which authenticate their cell lines regularly by DNA fingerprinting. After cell editing, we confirmed genomic integrity by Illumina Infinium GSAMD-24v2-0 and CNV analysis. We confirm identity by Sanger Sequencing of EZH1 variants. HEK293-T were not further authenticated since they were only used for lentiviral production, which was successfully achieved.
Mycoplasma contamination	All cell lines are tested once a month for negative for mycoplasma contamination.
Commonly misidentified lines<br>(See ICLAC register)	HEK293-T

Animals and other research organisms

Policy information about studies involving animals; ARRIVE guidelines recommended for reporting animal research, and Sex and Gender in Research

Laboratory animals	Wildtype embryos from White Leghorn chickens were used at stages ranging from HH14 to HH30.
Wild animals	This study did not include wild animals.
Reporting on sex	No sex is recorded for the chick embryos used in this study.
Field-collected samples	This study did not involve samples collected from the field.
Ethics oversight	No ethical approval is required for studies in chick embryos under the animal care guidelines of the EU (where these experiments were performed) since the embryos used in this study were all in early stages of embryonic development (between embryonic day 2 and 7).

Note that full information on the approval of the study protocol must also be provided in the manuscript.

ChIP-seq

Data deposition

☒ Confirm that both raw and final processed data have been deposited in a public database such as GEO.

☒ Confirm that you have deposited or provided access to graph files (e.g. BED files) for the called peaks.

Data access links<br>*May remain private before publication.*	https://www.ncbi.nlm.nih.gov/geo/query/acc.cgi?acc=GSE210465<br>Token to access: mtezuguepbonxyj
Files in database submission	GSM6430760 GFP, input, R1<br>GSM6430761 GFP, input, R2<br>GSM6430762 GFP, input, R3<br>GSM6430763 EZH1, WT, input, R1<br>GSM6430764 EZH1, WT, input, R2<br>GSM6430765 EZH1, WT, input, R3<br>GSM6430766 EZH1, A678G, input, R1<br>GSM6430767 EZH1, A678G, input, R2<br>GSM6430768 EZH1, A678G, input, R3<br>GSM6430769 GFP, H3K27me3, R1<br>GSM6430770 GFP, H3K27me3, R2<br>GSM6430771 GFP, H3K27me3, R3<br>GSM6430772 EZH1, WT, H3K27me3, R1<br>GSM6430773 EZH1, WT, H3K27me3, R2<br>GSM6430774 EZH1, WT, H3K27me3, R3<br>GSM6430775 EZH1, A678G, H3K27me3, R1

	GSM6430776 EZH1, A678G, H3K27me3, R2<br>GSM6430777 EZH1, A678G, H3K27me3, R3
Genome browser session (e.g. UCSC)	N/A

Methodology

Replicates	3 independent biological replicates per condition was performed:<br>GFP, input, R1<br>GFP, input, R2<br>GFP, input, R3<br>EZH1, WT, input, R1<br>EZH1, WT, input, R2<br>EZH1, WT, input, R3<br>EZH1, A678G, input, R1<br>EZH1, A678G, input, R2<br>EZH1, A678G, input, R3<br>GFP, H3K27me3, R1<br>GFP, H3K27me3, R2<br>GFP, H3K27me3, R3<br>EZH1, WT, H3K27me3, R1<br>EZH1, WT, H3K27me3, R2<br>EZH1, WT, H3K27me3, R3<br>EZH1, A678G, H3K27me3, R1<br>EZH1, A678G, H3K27me3, R2<br>EZH1, A678G, H3K27me3, R3
Sequencing depth	DNA libraries were sequenced at 40 million 150 bp pair-end reads per replicate in the NovaSeq 6000.
Antibodies	antiH3K27me3 EMD (Millipore 07-449)
Peak calling parameters	H3K27me3 peaks were called using MACS2 with parameters: --broad --keep-dup all -p 1e-5 --broad-cutoff 1e-5.
Data quality	4768 peaks were identified from GFP ReNcell H3K27me3 ChIP-seq data with p value 1e-5 cutoff.
Software	ChIP-seq signal was normalized to sequencing depth using deepTools: bamCoverage --normalizeUsing CPM71. ChIP-seq signal around peaks was computed and visualized using deepTools computeMatrix and plotHeatmap functions, respectively.

Flow Cytometry

Plots

Confirm that:

☒ The axis labels state the marker and fluorochrome used (e.g. CD4-FITC).

☒ The axis scales are clearly visible. Include numbers along axes only for bottom left plot of group (a 'group' is an analysis of identical markers).

☒ All plots are contour plots with outliers or pseudocolor plots.

☒ A numerical value for number of cells or percentage (with statistics) is provided.

Methodology

Sample preparation	For the immunostaining and flow analysis, cells were retrieved in Accutase, diluted with 1X DPBS, and centrifuged at 3000 x g, followed by resuspension and fixation in 1.6% paraformaldehyde for 30min at 37C with agitation. Cells were subsequently washed once with 1X DPBS, resuspended into FACS Buffer (1X DPBS containing 0.5% BSA (Jackson ImmunoResearch) and 0.05% sodium azide) and stored at 4C until ready for analysis. Cells were then permeabilized with saponin buffer (diluted to 1X in water; Biolegend, # 421022) and incubated for 1h in the following antibodies diluted in saponin buffer: anti-Ki67 (1:400; Cell Signaling #9449), anti-HuC/D (1:200; Invitrogen, A-21271), and anti-SOX2 (1:300; Cell Signaling, #3579). Cells were subsequently washed with saponin buffer and incubated for 1h with secondary antibodies goat anti-mouse IgG1-conjugated Alex 647, goat anti-mouse IgG2b-conjugated Alexa 488, and goat anti-rabbit conjugated brilliant violet 421 (each at 1:500; Jackson ImmunoResearch 115-605-205, 115-545-207, 111-675-144, respectively). Cells were subsequently washed and resuspended into FACS buffer.
Instrument	CytoFLEX flow cytometer (Beckman Coulter)
Software	FlowJo software program (BD).
Cell population abundance	Abundance of the cell populations are provided in the contour plots. Cells were not sorted.
Gating strategy	The starting cell populations was selected using the area of the following coordinates (FSC-A, SSC-A): (146, 192k), (146k, 370k), (291k,642k), (551k, 932k), (906k, 1.1M), (970k, 614k), (391k,112k), (192k,89k). These criteria selected 86+-3% [SEM]

of events of Day 0 samples, and 72+-2% [SEM] of events of Day 5 samples, such that the area selected excluded debris and dead/dying cells. Positive and negative cell staining was defined as a direct comparison of the Day 0 vs Day 5 timepoints, such that the earlier timepoint (Day 0) determined the gating, knowing that the cells were proliferative (Ki67+, SOX2+) and not yet terminally differentiated (HuCD-). The same gates, without modification, were applied to the Day 5 timepoint. Population groups and clustering were taken into consideration to be inclusive of the indicated group to provide appropriate gating as to not split a population.

☒ Tick this box to confirm that a figure exemplifying the gating strategy is provided in the Supplementary Information.
